# Alterations in Placental Inflammation-Related Gene Expression Partially Mediate the Effects of Prenatal Alcohol Consumption on Maternal Iron Homeostasis

**DOI:** 10.3390/nu15194105

**Published:** 2023-09-22

**Authors:** Jacqueline J. Masehi-Lano, Maya Deyssenroth, Sandra W. Jacobson, Joseph L. Jacobson, Christopher D. Molteno, Neil C. Dodge, Helen C. Wainwright, Ernesta M. Meintjes, Corina Lesseur, Haoxiang Cheng, Qian Li, Ke Hao, Jia Chen, R. Colin Carter

**Affiliations:** 1 Institute of Human Nutrition and Departments of Emergency Medicine and Pediatrics, Columbia University Vagelos College of Physicians and Surgeons, New York, NY 10032, USA; 2 Department of Environmental Health Sciences, Mailman School of Public Health, Columbia University, New York, NY 10032, USA; 3 Department of Psychiatry and Behavioral Neurosciences, Wayne State University School of Medicine, Detroit, MI 48201, USA; 4 Department of Human Biology, University of Cape Town Faculty of Health Sciences, Cape Town 7925, South Africa; 5 Department of Psychiatry and Mental Health, University of Cape Town Faculty of Health Sciences, Cape Town 7925, South Africa; 6 Department of Pathology, National Health Laboratory Service, Cape Town 7925, South Africa; helen.cecilia.wainwright@gmail.com; 7 Department of Environmental Medicine and Public Health, Icahn School of Medicine at Mount Sinai, New York, NY 10029, USA; 8 Department of Genetics and Genomic Sciences, Icahn School of Medicine at Mount Sinai, New York, NY 10029, USA

**Keywords:** fetal alcohol syndrome (FAS), fetal alcohol spectrum disorders (FASD), inflammation, iron deficiency (ID), iron deficiency anemia (IDA), iron metabolism, placenta, placental gene expression

## Abstract

Prenatal alcohol exposure (PAE) is associated with alterations in maternal and infant iron homeostasis that are consistent with changes seen in the setting of inflammation. We hypothesized that PAE leads to alterations in the placental expression of genes related to iron metabolism and inflammation that play functional roles in the teratogenic effects of alcohol on iron homeostasis. A total of 126 heavy-drinking women (≥1 oz (30 mL) absolute alcohol/day (~1.67 standard drinks/day) or women reporting binge drinking (≥2 drinks/occasion)) and 80 control women (<0.5 oz AA per day, no binging) in Cape Town, South Africa were interviewed prenatally regarding demographics, and alcohol, smoking, and drug use around conception and during pregnancy. Prenatal/maternal and infant hemoglobin and ferritin were measured. Whole-transcriptome RNA sequencing analysis was performed on flash-frozen transplacental tissue samples. Gene sets related to iron metabolism (*n* = 398) and inflammation (*n* = 467) were constructed by searching the Molecular Signatures Database for related ontology terms. Principal component analysis (PCA) yielded 59 factors for each theme. In multivariable regression models, PAE was related to 2 iron metabolism PCA factors (PCs) and 5 inflammation PCs, among which 2 iron metabolism and 4 inflammation factors were related to at least 1 key maternal or infant iron outcome. In causal inference analyses based on marginal structural models and the product method, the alterations in the expression profile of genes with functions in immune cell regulation, cytokine activity, angiogenesis, hematopoiesis, and ubiquitous cell processes appeared to partially mediate the relation of prenatal drinking frequency (days/week) around conception to a lower maternal hemoglobin-to-log(ferritin) ratio (proportion mediation = 51.35%). These findings suggest that placental inflammation may be partly responsible for the differences in alcohol-related iron homeostasis patterns between pregnant and non-pregnant adults.

## 1. Introduction

Fetal alcohol spectrum disorders (FASD) is the collective term for the broad range of congenital conditions caused by fetal exposure to alcohol during pregnancy [1,2,3]. FASD are characterized by neurocognitive deficits, structural brain abnormalities, pre- and postnatal growth restriction, and distinctive facial dysmorphology. Prenatal alcohol exposure (PAE) is the most common preventable cause of neurodevelopmental delay in children [4]. In the United States and Western Europe, prevalence estimates for FASD range from 2.0–5.0% of school-aged children, with even greater prevalence (13.6–20.9%) being estimated in Cape Town, South Africa [5,6]. Although more than five decades of research have documented the teratogenic effects of alcohol affecting every organ system [3,7,8,9], a recent United States Centers for Disease Control report documented that 11.9% of pregnant women reported current drinking [10].

In a prenatally recruited, prospective longitudinal cohort study of 126 heavy-drinking pregnant women and 80 abstaining/light-drinking controls in Cape Town, South Africa, alcohol consumption was related to higher maternal serum ferritin, a higher prevalence of prenatal anemia of inflammation, and higher maternal hepcidin, the chief negative regulator of iron bioavailability in circulation [11]. Prenatal alcohol consumption was also related to a reduced maternal hemoglobin-to-log(ferritin) ratio, suggesting an alcohol-related shift of iron sequestered into storage at the expense of erythropoiesis and other biological processes, such as placental iron transport. This shift of iron sequestered into storage at the expense of erythropoiesis is consistent with the iron homeostasis changes seen in the setting of inflammation [12,13]. Placental tissue sections stained for iron transport proteins revealed evidence of iron-restricted placental iron transport resulting from an insufficient maternal iron supply, as demonstrated by the association between PAE and a lower placental ferroportin-1 (FPN-1) to transferrin receptor-1 (TFR-1) ratio [11,14]. The shift of maternal iron into storage partially mediated a similar PAE-related decrease in neonatal hemoglobin-to-log(ferritin) ratio. This reduction in the neonatal hemoglobin-to-log(ferritin) ratio, in turn, partially mediated a PAE-related increase in iron deficiency anemia (IDA) at age 6.5 months. These findings confirmed those of a previous report in a similarly designed prospective longitudinal birth cohort, in which infants with heavy alcohol exposure had 3.6-times greater odds of developing IDA [15].

Taken together, these findings suggest that PAE-induced inflammation decreases the bioavailability of iron to the placenta and fetus and, in turn, leads to iron deficiency (ID) during infancy. Alcohol is known to be pro-inflammatory, and animal and human studies have demonstrated altered cytokine profiles in the mother and infant in the setting of maternal alcohol consumption [16,17,18,19]. Of note, the alcohol-related iron homeostasis changes in heavy-drinking pregnant women are quite different from what is seen in non-pregnant alcohol-consuming adults. In non-pregnant adults, heavy alcohol consumption leads to iron overload and decreased hepcidin [20]. Given the placenta’s critical roles in physiologic alterations during pregnancy, we hypothesized that alterations in the placental expression of genes related to iron metabolism and/or inflammation may contribute to the effects of PAE on maternal and infant iron homeostasis.

## 2. Materials and Methods

### 2.1. Sample

This study is a sub-study from the Cape Town longitudinal cohort described above; the recruitment and study design of the parent longitudinal cohort have been described in detail in this journal and elsewhere [11,21]. Briefly, pregnant women were recruited at initiation of antenatal care from two midwife obstetric units in Cape Town, South Africa (2011–2015). Alcohol consumption during pregnancy was ascertained prospectively using validated “timeline follow-back” interviews [22]. Women who averaged at least 1.0 oz absolute alcohol (AA) per day (1 Oz AA = 30 mL AA = 1.67 standard drinks) or reported at least two binge-drinking episodes (≥2.0 oz AA on one occasion) were invited to participate in the study. Women who reported abstaining or only minimal drinking (<0.5 oz AA per day) with no binge episodes were invited to participate as controls. All pregnant women were counseled to cease or reduce their alcohol intake as much as possible and offered referrals for substance use treatment. The maternal exclusion criteria for the parent study were age <18 years, multiple gestation pregnancy, human immunodeficiency virus (HIV) infection, and pharmacologic treatment for medical conditions such as epilepsy, diabetes, or cardiac problems. The infant exclusion criteria were major chromosomal abnormalities, seizures, neural tube defects, very low birthweight (<1500 g), and extreme prematurity (<32 weeks gestation). Gene expression assays (described below) were conducted only on placenta samples flash-frozen within 72 h of delivery from women not meeting the following additional exclusion criteria known to affect placental development: regular methamphetamine use (at least monthly), delivery <32 weeks gestation, low birthweight (<2500 g), and development of hypertension, preeclampsia, or syphilis during pregnancy. Based on prior analyses that integrated infant and maternal gene expression and genotyping data, one sample with >5% estimated maternal cell contamination was identified and removed from the current study. Thus, the final sample included 35 heavy-drinking and 33 control women and their infants (see Supplementary File S1, Figure S1 for a sample selection flow diagram).

### 2.2. Ascertainment of Maternal Alcohol, Cigarette Smoking, and Drug Use

In “timeline follow-back” interviews administered at recruitment and again at 4 and 12 weeks thereafter, each woman was asked about her daily drinking during the previous 2 weeks, with reference to specific daily activities to assist in recall [22]. She was also asked to report her drinking during any weeks when she drank greater quantities since the last visit. At the recruitment visit, each woman was asked about her drinking during a typical 2-week period around the time of conception. Separately for periconceptional drinking and drinking averaged across pregnancy, three different summary alcohol measures were constructed: oz AA/day (1 oz = 30 mL), oz AA/drinking occasion, and frequency of drinking (days/week).

### 2.3. Demographics and Potential Covariates

Participants were also interviewed regarding their demographic background, including age, gravidity, socioeconomic status (SES) [23], and education. Each participant was asked to report the frequency of smoking and illicit drug use (cocaine, methamphetamine, opiates, methaqualone, and marijuana) throughout pregnancy. Maternal self-reports of drug use were validated by testing urine specimens using the AccuTest^TM^ 6 + 2 drugs of abuse panel test (DTA Pty Ltd., Cape Town, South Africa), an immunochemical assay that detects metabolites of drugs commonly used in the community (amphetamines, cocaine, methaqualone, methamphetamine, opiates, and marijuana) [8]. 

### 2.4. Dietary, Hematological, and Biochemical Iron Indicators

Multi-pass 24-h dietary recall interviews were administered at each antenatal study visit to assess the average daily iron and energy intakes, which were processed with FoodFinder3 (South African Medical Research Council) [11]. Serum ferritin concentration and complete blood count analyses, including hemoglobin concentration, were performed at recruitment and 12 weeks later and averaged; blood from the infants was assayed at ages 2 weeks and 6 months postpartum (corrected for prematurity). The 6-month infant samples were also analyzed for soluble transferrin receptor (sTfR) and C-reactive protein (CRP). Infants were diagnosed with ID if they had (1) serum ferritin < 12 μg; or (2) in cases with CRP > 5 mg/L, elevated sTfR > 8.3 mg/L for infants at 6 months [24,25,26]. Infants were diagnosed with IDA if they had (1) hemoglobin < 11 g/L, (2) red cell distribution width > 15.0%, and either (3) mean corpuscular volume ≤ 70.0 or mean corpuscular hemoglobin ≤ 23.0 [26]. Infants were determined to have anemia of other causes and excluded from all the analyses involving ID status if they exhibited hemoglobin < 11 g/L but did not meet the ID criteria. Maternal urinary hepcidin:creatinine was measured as a proxy for the blood hepcidin concentration via competitive enzyme-linked immunosorbent assay in morning urine samples [27]. To assess the balance of iron in storage compared to the iron available for erythropoiesis in the mothers and infants, a hemoglobin:log(ferritin) variable was calculated. 

### 2.5. Placental Gene Expression

Placenta samples were collected using a validated protocol that would minimize perturbations in gene expression during the period between delivery and sample collection [28]. Briefly, the collected placentas were immediately stored in a commercial refrigerator (~2 to 3 °C) and transported to University of Cape Town (UCT) by van in a cooler box (also ~2 to 3 °C) by research staff. Co-author H. Wainwright, MD, a senior placenta pathologist at the UCT Faculty of Health Sciences, conducted histopathology examinations of the placentas and rinsed and flash-froze (−80 °C) transplacental core tissue samples from the center of each of four cotyledons. To be included in ribonucleic acid sequencing (RNA-Seq) analyses, placental samples needed to be flash-frozen within 72 h of delivery to prevent RNA breakdown (range = 4–72 h). The samples were shipped with a continuous cold chain to the laboratory of co-author J. Chen in New York City, USA, where RNA was extracted in 2020 from placental tissue that was homogenized using the Maxwell 16 LEV simplyRNA Tissue Kit (Promega, #AS1280; Madison, WI, USA). Ribosomal RNA depletion library preparation and pair-end RNA sequencing were conducted. The sequencing was performed using DNBseq™ technology (MGI, Shenzhen, China); all samples had >46 million reads sequenced (average: 48.5 million). The RNA-Seq reads (fastq files) were characterized using MultiQC [29]. The reads were quality-filtered (90% of bases quality score > Q20) and aligned and quantified using STAR [30] and GenCode v33 annotation of the human genome assembly GRCh38. Technical variables that can influence the gene expression profiles were examined using VariancePartion [31], and the ComBat-seq function from the SVA R packages [32] was used to adjust the gene counts for the time to sample freezing as a categorical variable (tertiles).

### 2.6. Curated Gene List and Principal Component Analysis (PCA)

The analysis plan is delineated in Supplementary File S1, Figure S2. Sets of candidate genes related to iron metabolism (*n* = 503) and inflammation (*n* = 600) were curated by searching the Molecular Signatures Database (MSigDB) with iron metabolism- and inflammation-related ontology terms [33,34]. Genes that were expressed in <50% of placental samples were removed, resulting in gene sets of 398 iron metabolism genes and 467 inflammation genes. The gene sets and the ontology terms used are listed in Supplementary File S2 “Gene Sets”.

Using SPSS (v.28; IBM), dimension reduction was employed within each gene set (i.e., iron metabolism- and inflammation-related genes) using principal component analysis (PCA) [35]. The PCA factor (PC) scores were based on regression models, and factors with an eigenvalue ≥1 were extracted and retained for subsequent analyses. No rotation was applied to minimize the correlation between factors. Supplementary File S1, Figure S3 depicts scree plots of the PCA analyses for both gene sets. The first 59 PCs derived from the iron metabolism-related gene set had eigenvalues ≥1 and explained 99.49% of the variance. The first 59 PCs derived from the inflammation-related gene set had eigenvalues ≥1 and explained 99.43% of the variance. To evaluate the potential physiologic roles of PCs of interest, the functions of genes related to the factors of interest with |*r*| ≥ 0.2 were assessed using the genecards.org database [36] and PubMed literature searches.

### 2.7. Statistical Analyses

All statistical analyses were 2-sided (α = 0.05) using SPSS (v.27.0; IBM) or Stata (v.17.0; StataCorp). All variables were examined for the normality of the distribution; the oz AA/day and ferritin concentrations were log-transformed due to skewness (>3.0). Pearson correlation analyses were used to examine the potential associations between the PAE variables and the placental gene expression PCs, and between the PCs (i.e., those related to PAE) and the maternal and infant outcome measures. Next, multivariable regression models were constructed to examine the relations between PAE and the PCs, and between the PCs and outcomes, adjusting for potential confounders. In PAE–PC multivariable regression models, potential confounders included the time from delivery to placental sample freezing, weeks gestation at delivery, and any potential covariates (maternal age, SES, maternal education, gravidity, prenatal cigarettes/day, days/month maternal marijuana use, weeks formula feeding) related to a given factor at *p* ≤ 0.10 (see Supplementary File S3 “Pearson Correlations”). In the PC–outcome regression models, the same variables related to the factor were controlled for, as well as potential confounders for the maternal and infant iron outcomes identified in previous analyses in this cohort [11]: maternal ferritin and maternal hemoglobin:log(ferritin): maternal age; maternal urinary hepcidin:creatinine: maternal iron supplementation (yes vs. no); neonatal hemoglobin, ferritin, and hemoglobin:log(ferritin): age at time of blood draw; hemoglobin at 6 months: age at time of blood draw; ID at 6 months (yes vs. no): maternal iron supplementation (yes vs. no), number of weeks infant was formula fed; IDA at 6 months (yes vs. no): maternal iron supplementation (yes vs. no), maternal gravidity, and number of weeks infant was formula fed.

To examine the hypothesis that the relations of PAE to maternal and infant iron measures are mediated by the placental expression profiles of iron metabolism- and/or inflammation–related genes, causal inference analyses were conducted based on marginal structural models and the product method (paramed package, Stata) [37,38]. The analyses included potential confounders from the relevant PAE–mediator and mediator–outcome regression models described above.

## 3. Results

### 3.1. Sample Characteristics

In this sample, both heavy-drinking and control women were socioeconomically disadvantaged and poorly educated (Table 1). On average, mothers in the heavy-drinking group were 4.2 years older than the controls. Women are prescribed iron supplementation as part of the standard prenatal care in South Africa (67 mg elemental iron), and a greater percentage (91.4%) of the heavy-drinking pregnant women reported taking their prescribed iron supplements on most days compared to the control women (71.9%). The heavy-drinking women averaged 6.8 standard drinks/occasion (1 oz AA = 1.67 standard drinks) on 2.2 days/week around the time of conception and 7.2 standard drinks/occasion on 1.4 days/week averaged across pregnancy. This pattern is consistent with binge-drinking on weekends. Almost one in five heavy-drinking women met the criteria for alcohol dependence [39], while no control women met the criteria for dependence. All but 4 control women abstained from drinking during pregnancy, and none binge drank; 1 woman reported 1 drink during pregnancy, another 1 drink on 2 occasions, another 3 drinks on 1 occasion, and another 2-3 drinks 1-2 times per month. Cigarette smoking was prevalent, with two-thirds of both the heavy-drinking and control women reporting smoking. However, the number of cigarettes smoked per day was similar and generally low for both groups. No woman reported smoking >1.0 pack (20 cigarettes/day). Approximately one-tenth of both groups reported marijuana use, with the heavy-drinking women reporting smoking use on 10 days/month vs. 4 days/month among the control women. On average, the infants in both groups were born full-term, and small-for-gestational-age (SGA) status was common in both groups (almost one-third of the heavily exposed and almost one-fifth of the control infants). As previously reported [11], the heavy-drinking pregnant women had higher serum ferritin, hemoglobin:log(ferritin), and urinary hepcidin:creatinine. In the larger parent study [11], the continuous measures of PAE were related to higher ferritin and lower hemoglobin:log(ferritin) at age 2 weeks and lower hemoglobin and higher prevalence of ID and IDA at 6.5 months. In this smaller subset for whom placental gene expression data were available, only a trend for lower neonatal hemoglobin among the heavily exposed infants was seen in the between-group comparisons.

### 3.2. Relation of PAE to PCs

The associations between PAE and the iron metabolism and inflammation PCs with *p* < 0.10 are presented in Table 2 (for the full univariable results, please see Supplementary File S3 “Pearson Correlations”). Of the 59 iron metabolism PCs, 3 (PCs 8, 44, 59) were related to a PAE measure in the univariable correlation analyses. In multivariable models adjusting for covariates, 2 (PCs 8, 18) were related to measures of PAE averaged across pregnancy. Of the 59 inflammation PCs, 8 (PCs 5, 10, 11, 12, 22, 35, 40, 45) were related to a PAE measure in the univariable analyses. In multivariable models adjusting for covariates, 3 (PCs 5, 10, 35) were related to measures of PAE both around conception and averaged across pregnancy, and 2 (PCs 22, 40) were related only to measures of PAE around conception.

### 3.3. Relation of PCs to Maternal and Infant Iron Outcomes

Table 3 presents the regression models examining the relations between those iron metabolism and inflammation PCs related to PAE at *p* < 0.10 in Table 2, and the maternal and infant iron outcomes (for the full univariable results, please see Supplementary File S3 “Pearson Correlations”). No iron metabolism PC was related to maternal hemoglobin:log(ferritin). Two iron metabolism factors (PCs 20, 44) were related to maternal urinary hepcidin:creatinine in univariable analyses, although only PC 20 was associated with this outcome when adjusting for covariates. No iron metabolism PC was related to neonatal iron measures. At 6.5 months, iron metabolism PC 18 was related to infant hemoglobin in both univariable and multivariable analyses; PC 13 was related to ID in univariable but not multivariable analyses. Iron metabolism PC 18 was related to 6.5-month IDA in multivariable analyses. Inflammation PC 10 was related to maternal ferritin and hemoglobin:log(ferritin) in both univariable and multivariable analyses. No inflammation PC was related to any neonatal iron measure. At age 6.5 months, inflammation PC 12 was related to infant ID and PC 22 to infant IDA in both univariable and multivariable analyses. Inflammation PC 45 was related to 6.5-month infant hemoglobin in multivariable analyses.

### 3.4. Causal Inference Analyses Examining PCs as Potential Mediators of the Relations of PAE to Maternal and Infant Iron Outcomes

**Figure 1:**
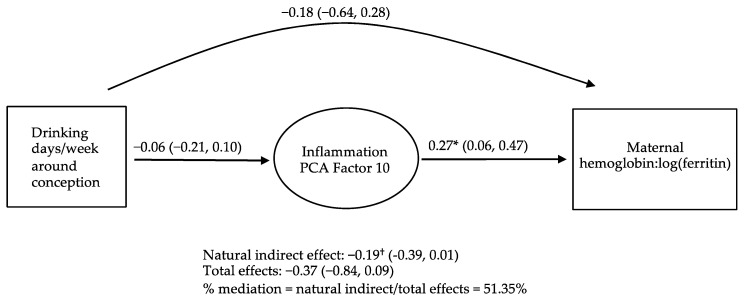
Causal mediation analyses. Directed acyclic graph demonstrating inflammation principal component analysis (PCA) factor 10 as a potential mediator of the relation between drinking frequency (modeled as increasing from 0 to 3 days/week) around conception and maternal hemoglobin-to-log(ferritin) ratio. Values are unstandardized B (95% confidence intervals) from marginal structural models using the product method [37,38] adjusting for the time from delivery to placental sample freezing, weeks gestation at delivery, maternal age at conception, and socioeconomic status. † *p* ≤ 0.10; * *p* ≤ 0.05.

Causal inference analyses were conducted on triads where PAE was related to a PC that was also related to a PAE-related maternal or infant iron outcome (Supplementary File S1, Table S1). As shown in Figure 1, a trend (*p* < 0.10) was seen for decreased placental expression of inflammation PC 10 to mediate 51.35% of the association of the drinking frequency around conception with decreased maternal hemoglobin:log(ferritin). In sensitivity analyses, causal inference analysis was conducted with PC 10 as the outcome and maternal hemoglobin:log(ferritin) as the mediator, and no evidence of mediation was seen. No evidence was seen for the other PAE–PC–iron outcome triads.

### 3.5. Functions of Genes Contributing to Inflammation PC 10

**Figure 2:**
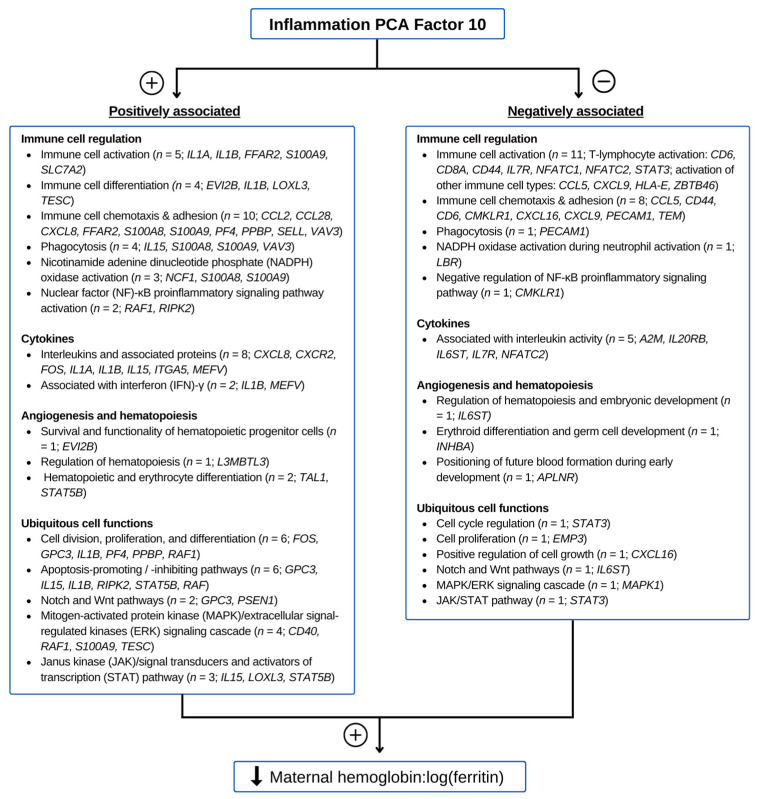
Major functional themes of genes associated with inflammation-related principal component analysis (PCA) factor 10.

The functions of the genes related to inflammation PC 10 with a Pearson correlation |r| ≥ 0.2 (see Supplementary File S3 “Pearson Correlations”) are described in Supplementary File S1, Supplementary S1, and they are categorized in Figure 2 from canvassing genecards.org [36] and PubMed. Angiogenesis and hematopoiesis represented a prominent category; 4 placental genes involved in hematopoiesis were positively associated with inflammation PC 10, and 3 genes related to hematopoiesis and angiogenesis were negatively associated with the factor. Of the placental genes involved in immunity that were positively related to the factor, 5 have immune cell activation functions, 4 are involved in immune cell differentiation, 10 are linked to immune cell chemotaxis and adhesion, 4 play critical roles in phagocytosis, 3 participate in nicotinamide adenine dinucleotide phosphate (NADPH) oxidase activation for superoxide production, and 2 are in the nuclear factor (NF)-κB proinflammatory signaling pathway. Within the placental genes negatively related to the factor, 11 are involved immune cell activation, 8 are involved in immune cell chemotaxis and adhesion, 1 plays roles in phagocytosis, 1 participates in NAPDH oxidase activation, and 1 plays a role in negative regulation of the NF-κB pathway. Another largely represented functional theme was cytokine regulation. Within the class of inflammatory genes that were positively associated with inflammation PC 10, 7 relate to cytokines and 2 to the regulation of interferon (IFN)-γ; most notably, genes *IL1A*, *IL1B*, *CXCL8*, and *IL15* respectively encode the cytokines interleukin (IL)-1α and IL-1β, which induce hepcidin production; IL-8 induces ferritin; and IL-15 mediates the suppression of erythropoiesis. Five genes related to cytokine activity, including *A2M*, *IL20RB*, *IL6ST*, and *IL7R*, were negatively associated with the factor. Of the placental genes representing ubiquitous cell functions that were positively related to inflammation PC 10, 6 play roles in cell division, proliferation, and differentiation; six function in apoptosis-promoting or -inhibiting pathways; 2 are involved in the Notch and Wnt pathways; 4 participate in the mitogen-activated protein kinase (MAPK)/extracellular signal-regulated kinases (ERK) signaling cascade; and 3 are involved in the Janus kinase (JAK)/signal transducers and activators of transcription (STAT) signaling pathway. 3 placental genes involved in ubiquitous cell functions were negatively related to inflammation PC 10: 1 in the Notch pathway, 1 in the MAPK/ERK signaling cascade, and 1 in the JAK/STAT pathway. Among these genes, *STAT3* is notable for encoding the transcription activator that induces hepatic hepcidin expression during inflammation [41,42].

## 4. Discussion

In this examination of placental gene expression in a prospective longitudinal cohort of mother–infant pairs in Cape Town, South Africa, maternal alcohol consumption was associated with changes in the iron metabolism- and inflammation-related gene expression profiles in the placenta. Given the critical role of the placenta as the key interface between the mother and fetus in both altering maternal physiology and the delivery of oxygen and nutrients to support fetal development, these changes may have functional significance in the development of FASD. For inflammation, these changes appeared to have possible mechanistic significance in a previously reported alcohol-induced shift of maternal iron into storage at the expense of hematopoiesis and placental iron transport [11]. To the best of our knowledge, this is the first human study to examine the impact of maternal alcohol consumption on the placental expression of genes related to iron metabolism and inflammation, and their potential functional significance.

Heavy alcohol consumption has been associated with a pro-inflammatory state in both humans and animals [18,43]. Findings include PAE-related increases in maternal and fetal IL-6, IL-1β, and tumor necrosis factor (TNF)-α, the chief inflammatory stimulators of hepcidin and ferritin [19]. In a prospective longitudinal birth cohort in Ukraine, prenatal alcohol consumption was related to alterations in maternal cytokine profiles [16]. Differential cytokine profiles were associated with neurodevelopmental delay, although they failed to distinguish between alcohol exposed and unexposed children with such delays. In other analyses from this cohort, elevations in specific cytokines decreased the odds of having a child with an FASD [17], and PAE was associated with alterations in the cytokine profiles of toddlers [44]. PAE has previously been shown to alter glucocorticoid receptor and inflammatory signaling [45], as seen in a recent prospective birth cohort in Australia, where PAE was generally low to moderate [46]. In a prospective birth cohort in Ireland, in which the majority of women abstained or drank at low–moderate levels, a more pro-inflammatory diet was associated with larger placental weight [47]. By contrast, alcohol consumption was associated with smaller placental weight and a lower placenta-to-birthweight ratio, as has been previously demonstrated [8,48]. Further studies are needed to examine the potential functional roles of inflammatory alterations in the placenta in FASD, neurobehavioral deficits and alterations in child immune function.

As described above, prenatal alcohol consumption was previously reported to be associated with alterations in maternal iron homeostasis that are consistent with a pro-inflammatory state in this cohort [11]. These alterations are notably different from the changes seen in non-pregnant heavy-drinking adults [20]. The findings presented in the current study suggest that this discrepancy may be due, in part, to inflammatory changes in the placenta that alter maternal physiology. Of note, these maternal iron homeostasis alterations may have functional significance in FASD, as they mediated similar alterations in the neonate, which, in turn, mediated a PAE-related increase in infant iron deficiency anemia. Furthermore, PAE-related elevations in maternal ferritin appeared to exacerbate the effects of PAE on infants’ recognition memory and processing speed, and they partially mediated PAE-related reductions in head circumference, weight, and length [21]. Thus, placental inflammation may represent a new target for interventions aimed at mitigating the teratogenic effects of alcohol. A growing body of literature has implicated neuroinflammation as an important mechanism in alcohol-induced damage to the developing brain, although the role of placental inflammation in fetal neuroinflammation remains largely unknown. Beyond targeting placental inflammation, maximizing maternal–fetal iron stores may potentially overcome inflammation-induced functional iron deficiency and mitigate some teratogenic effects of alcohol, as has been demonstrated in animal models by Smith and colleagues [18,49,50].

The functions of genes that contributed to the inflammation PCA factor with evidence of possible mediation in the PAE-related shift of maternal iron into storage may shed light on the potential mechanisms underlying these findings. Firstly, this factor was negatively associated with alcohol and positively associated with maternal hemoglobin:log(ferritin). Consistent with this, *STAT3*, the protein which is integral to the IL-6-mediated induction of hepcidin, was negatively related to the factor. *IL15*, which encodes the cytokine IL-15, was positively related to the factor. IL-15 contributes to the development of anemia in mediating the suppression of erythropoiesis by IFN-γ [51]. Other functions negatively related to the factor include immune cell activation (11 genes), immune cell chemotaxis and adhesion (8 genes), NADPH oxidase activation in neutrophils (*LBR*), and hematopoiesis (3 genes). Some genes with functions related to increased hepcidin and ferritin were positively related to the factor and thus were unlikely to have contributed to the shift in iron into storage seen in the mother. Instead, these genes may have been upregulated by the placenta as a fetal response to alcohol and/or iron restriction. These genes included *IL1A* and *IL1B*, which encode the pro-hepcidin cytokines IL-1α and IL-1β [52]. Up-regulation of IL-1β induces iron uptake into macrophages, promotes the destruction of erythrocytes, stimulates ferritin biosynthesis, thereby increasing the amount of iron in storage, and inhibits FPN-1, thus blocking cellular iron export. Similarly, IL-8 induces serum ferritin [53], and inflammation PC 10 was positively associated with *CXCL8*, which encodes IL-8, *CXCR2*, which encodes the IL-8 receptor, and *FFAR2*, which induces IL-8 secretion.

This study had several limitations common to prospective longitudinal studies of PAE. First, some children were not able to attend either the 2-week or 6-month visit, thus reducing the sample size for several infant outcomes. Although the mothers in the study were recruited from the same socioeconomically deprived community, some degree of residual confounding is possible due to unmeasured environmental, dietary, and genetic influences on postnatal growth and iron homeostasis. The precision and accuracy of estimating true PAE may be affected by maternal and fetal alcohol metabolism due to genetics, body size, and metabolic activity. However, the differences between the estimated and true PAE measures are likely minor, as maternal reports of drinking in the Cape Town community have been previously validated by levels of fatty acid ethyl esters in meconium specimens. As RNA-Seq data from bulk tissue were used, the expression profiles examined were derived from multiple placental cell types. Given the small sample size of the cohort (*N* = 68), this study was not powered to detect statistically significant interaction effects of PAE and PCs, and multiple testing correction was not performed. Given the possibility of type-II (false negative) errors, the lack of evidence of mediation by iron metabolism- and inflammation-related PCs in relation to the maternal and infant iron outcomes beyond maternal hemoglobin:log(ferritin) in no way suggests that the PAE-related changes in the placental gene expression profiles that were observed do not have functional roles. Rather, such roles may have been difficult to detect in the cohort’s placental gene expression data patterns. The causal inference approach used assumes that the placental gene expression profiles at delivery were representative of the placental gene expression when the maternal iron assays were measured. Thus, the impact of placental gene expression on maternal physiology for genes where the expression would have changed over gestation could not be assessed in this study. To address this potential issue, for the inflammation PC 10 causal inference analyses, the model was first run in the hypothesized direction with the PC as the mediator and maternal hemoglobin:log(ferritin) as the outcome. The model was then run again with maternal hemoglobin:log(ferritin) as the mediator and the factor as the outcome, and no evidence of mediation in this model was seen. Another limitation of this study is that the MSigDB used to construct the iron metabolism and inflammation gene sets is limited to genes and functions that have been previously studied, which may be biased toward genes and functional roles related to topics that are heavily studied, e.g., cancer, and away from topics that are relatively less studied, e.g., teratology. However, these data are continuously being updated by outside contributors.

## 5. Conclusions

PAE was related to alterations in placental gene expression profiles in two areas hypothesized to have mechanistic importance in FASDs: iron metabolism and inflammation. Furthermore, alterations in inflammation-related gene expression appeared to have potential functional significance in an alcohol-related shift of iron into storage in the mother, which may explain why this is seen in heavy-drinking pregnant women but not in heavy-drinking non-pregnant adults. Further research is needed to elucidate the mechanisms by which alterations in placental gene expression may contribute to the effects of alcohol on maternal physiology, e.g., secretion of placental extracellular vesicles. Such understanding may inform new intervention strategies targeting placental inflammation.

## Figures and Tables

**Table 1:** Sample characteristics.

	Controls (*n* = 35)	Heavy-Drinking Mothers (*n* = 33)	
	*M* (SD) or *n* (%)	*M* (SD) or *n* (%)	*p* ^a^
Maternal characteristics			
Age at conception (years)	25.1 (4.9)	29.3 (5.9)	0.002
Gravidity (no. previous pregnancies (%))	1.4 (1.2)	2.2 (1.5)	0.014
Education (years of school completed)	10.1 (1.4)	9.5 (1.2)	0.089
Socioeconomic status [23]	22.1 (6.9)	19.7 (6.0)	0.066
No. (%) taking maternal prenatal iron supplementation	23 (71.9)	32 (91.4)	0.037
Alcohol and drug use			
Around conception:			
Oz AA/day	0.0 (0.0)	1.4 (1.0)	<0.001
Oz AA/drinking day	0.1 (0.3)	4.1 (2.4)	<0.001
Drinking days/week	0.0 (0.1)	2.2 (1.2)	<0.001
Averaged across pregnancy:			
Oz AA/day	0.0 (0.1)	0.8 (0.7)	<0.001
Oz AA/drinking day	0.1 (0.5)	4.3 (1.7)	<0.001
Drinking days/week	0.0 (0.0)	1.4 (1.0)	<0.001
Current alcohol dependence ^b^	0 (0.0)	6 (18.8)	0.011
No. (%) reporting cigarette smoking	22 (66.7)	24 (68.6)	0.065
Cigarettes/day among smokers	5.8 (4.3)	7.0 (4.3)	0.352
No. (%) reporting marijuana use	4 (12.1)	4 (11.4)	0.929
Marijuana days/month among users	4.0 (4.7)	9.7 (9.0)	0.047
No. (%) reporting methamphetamine use	11 (15.5)	12 (13.8)	0.763
Methamphetamine days/month among users	4.2 (6.6)	7.5 (7.1)	0.525
Maternal iron measures			
Ferritin (logged ug/L values)	2.4 (0.5)	3.0 (0.7)	<0.001
Hemoglobin (g/dL):log(ferritin ug/L)	4.6 (0.8)	4.0 (0.8)	0.002
Urinary hepcidin (ng):creatinine (mg)	26.5 (5.4)	24.9 (6.3)	0.276
Infant characteristics			
Weeks of gestation at delivery	39.3 (1.7)	39.2 (1.8)	0.440
Sex, no. female (%)	14 (42.4)	13 (37.1)	0.656
Birthweight (g)	3100.9 (496.1)	2964.0 (532.2)	0.139
No. (%) small for gestational age ^c^	6 (18.2)	11 (31.4)	0.207
Neonatal iron measures			
Hemoglobin (g/dL) ^d^	15.3 (2.6)	14.9 (1.8)	0.219
Ferritin (logged ug/L values) ^e^	5.2 (0.7)	5.3 (0.6)	0.156
Hemoglobin (g/dL):log(ferritin) ^f^	3.0 (0.5)	2.8 (0.5)	0.088
6.5-month infant iron measures			
Hemoglobin (g/dL)	10.8 (1.0)	10.7 (1.2)	0.314
Iron deficiency (yes = 1, no = 0)	9 (27.3)	12 (34.3)	0.532
Iron deficiency anemia (yes = 1, no = 0)	4 (12.1)	7 (20.0)	0.378

AA = absolute alcohol; 1 Oz AA = 30 mL AA = 1.67 standard drinks. ^a^ From independent samples *t*-tests for continuous outcomes, chi-square for binary outcomes. ^b^ From the Structured Clinical Interview for Diagnostic and Statistical Manual of Mental Disorders, 5th edition [39]; missing for 2 control and 3 heavy-drinking mothers. ^c^ Defined as birthweight <10th percentile for gestational age [40]. ^d^ Missing for 6 control and 6 heavily exposed infants. ^e^ Missing for 5 control and 5 heavily exposed infants. ^f^ Missing for 6 control and 8 heavily exposed infants.

**Table 2:** Association of prenatal alcohol exposure with placental iron metabolism- and inflammation-related gene expression principal component analysis (PCA) factors.

**Iron Metabolism PCA Factor:**	**Factor 8**		**Factor 11 ^a^**		**Factor 13 ^b,c^**		**Factor 18 ^d^**		**Factor 20 ^e^**		**Factor 42**	
	***r***	***ß***	***r***	***ß***	***r***	***ß***	***r***	***ß***	***r***	***ß***	***r***	***ß***
Alcohol use around conception:												
Oz AA/day (logged values)	-	-	-	-	-	-	-	-	0.22 †	0.19	0.22 †	0.22 †
Oz AA/drinking day	−0.21 †	−0.22 †	-	-	-	-	-	-	-	-	0.22 †	0.22 †
Drinking days/week	-	-	−0.23 †	−0.23 †	-	-	-	-	-	-	-	-
Alcohol use across pregnancy:												
Oz AA/day (logged values)	-	-	-	-	-	-	−0.20 †	−0.27 *	0.22 †	0.18	-	-
Oz AA/drinking day	−0.28 *	−0.27 *	-	-	0.23 †	0.14	-	-	-	-	-	-
Drinking days/week	-	-	-	-	-	-	−0.20 †	−0.26 *	0.20 †	0.15	-	-
**Iron Metabolism PCA Factor:**	**Factor 44 ^c,f^**		**Factor 49**		**Factor 59 ^f^**							
	***r***	***ß***	***r***	***ß***	***r***	***ß***						
Alcohol use around conception:												
Oz AA/day (logged values)	-	-	-	-	-	-						
Oz AA/drinking day	-	-	-	-	-	-						
Drinking days/week	−0.25 *	−0.21 †	-	-	-	-						
Alcohol use across pregnancy:												
Oz AA/day (logged values)	-	-	-	-	−0.20 †	−0.13						
Oz AA/drinking day	-	-	-	-	-	-						
Drinking days/week	-	-	0.20 †	0.19	−0.25 *	−0.19						
**Inflammation PCA Factor:**	**Factor 5**		**Factor 10 ^e^**		**Factor 12**		**Factor 22 ^d^**		**Factor 34 ^d^**		**Factor 35**	
	***r***	***ß***	***r***	***ß***	***r***	***ß***	***r***	***ß***	***r***		***r***	***ß***
Alcohol use around conception:												
Oz AA/day (logged values)	-	-	−0.29 *	−0.28 *	-	-	0.26 *	0.24 †	-	-	−0.29 *	−0.25 *
Oz AA/drinking day	−0.26 *	−0.27 *	−0.30 **	−0.29 *	-	-	0.32 **	0.29 *	-	-	−0.26 *	−0.21 †
Drinking days/week	-	-	−0.32 **	−0.30 **	0.25 *	0.23 †	-	-	-	-	−0.31 **	−0.27 *
Alcohol use across pregnancy:									-	-		
Oz AA/day (logged values)	-	-	−0.24 *	−0.22 †	0.21 †	0.19	-	-	-	-	−0.35 **	−0.31 **
Oz AA/drinking day	−0.25 *	−0.27 *	−0.27 *	−0.24 *	-	-	-	-	0.22 †	0.20 †	−0.28 *	−0.25 *
Drinking days/week	-	-	−0.21 †	−0.19	0.24 *	0.22 †	-	-	-	-	−0.38 ***	−0.34 **
**Inflammation PCA Factor:**	**Factor 40**		**Factor 45**		**Factor 46**		**Factor 57**					
	***r***	***ß***	***r***	***ß***	***r***	***ß***	***r***	***ß***				
Alcohol use around conception:												
Oz AA/day (logged values)	0.26 *	0.26 *	0.28 *	0.23 †	-	-	-	-				
Oz AA/drinking day	0.22 †	0.22 †	0.28 *	0.23 †	−0.22 †	−0.22 †	-	-				
Drinking days/week	0.22 †	0.21 †	0.24 *	0.19	-	-	-	-				
Alcohol use across pregnancy:												
Oz AA/day (logged values)	0.21 †	0.20	0.24 *	0.18	-	-	-	-				
Oz AA/drinking day	-	-	0.23 †	0.17	-	-	-	-				
Drinking days/week	0.21 †	0.20	-	-	-	-	0.23 †	0.16				

† *p* ≤ 0.10; * *p* ≤ 0.05; ** *p* ≤ 0.01; *** *p* ≤ 0.001. AA = Oz absolute alcohol (1 oz = 30 mL); PCA = principal component analysis. *r* = Pearson correlation coefficient (N.B.: only values for which *p* < 0.10 are shown); *ß* = standardized regression coefficient adjusting for the time from delivery to placenta sample freezing, weeks’ gestation at delivery, and the following where noted: ^a^ no. prenatal cigarettes/day, ^b^ maternal age, ^c^ days/month prenatal marijuana use, ^d^ maternal education (highest grade completed), ^e^ socioeconomic status [23], and ^f^ gravidity. AA = Oz absolute alcohol (1 oz = 30 mL).

**Table 3:** Relations of prenatal alcohol exposure-related placental iron metabolism- and inflammation-related gene expression principal component analysis (PCA) factors to maternal and infant iron measures previously shown to be related to maternal alcohol consumption [21].

**Iron Metabolism PCA Factor:**	**Factor 8**		**Factor 11 ^a^**		**Factor 13 ^b,c^**		**Factor 18 ^d^**		**Factor 20 ^e^**	
	***r***	***ß***	***r***	***ß***	***r***	***ß***	***r***	***ß***	***r***	***ß***
Maternal iron measures:										
Ferritin (logged ug/L values) ^b^	-	-	-	-	-	-	-	-	-	-
Hemoglobin (g/dL):log(ferritin ug/L) ^b^	-	-	-	-	-	-	-	-	-	-
Urinary hepcidin (ng):creatinine (mg) ^g^	-	-	−0.31 †	−0.29	-	-	-	-	−0.38 *	−0.44 *
Neonatal iron measures:										
Hemoglobin (g/dL) ^h^	-	-	-	-	-	-	−0.23†	−0.19	-	-
Ferritin (logged ug/L values) ^h^	-	-	-	-	-	-	-	-	-	-
Hemoglobin (g/dL):log(ferritin) ^h^	-	-	-	-	-	-	-	-	-	-
6.5-month infant iron measures:										
Hemoglobin (g/dL) ^h^	-	-	-	-	-	-	0.24 *	0.28 *	-	-
Iron deficiency (Yes = 1, No = 0) ^g,i^	-	-	-	-	0.31 **	0.27 †	-	-	-	-
Iron deficiency anemia (Yes = 1, No = 0) ^f,g,i^	-	-	-	-	0.21 †	0.18	−0.22 †	−0.32 *	-	-
**Iron Metabolism PCA Factor:**	**Factor 42**		**Factor 44 ^c,f^**		**Factor 49**		**Factor 59 ^f^**			
	***r***	***ß***	***r***	***ß***	***r***	***ß***	***r***	***ß***		
Maternal iron measures:										
Ferritin (logged ug/L values) ^b^	-	-	-	-	-	-	-	-		
Hemoglobin (g/dL):log(ferritin ug/L) ^b^	-	-	-	-	-	-	-	-		
Urinary hepcidin (ng):creatinine (mg) ^g^	-	-	−0.39 *	−0.36 †	-	-	-	-		
Neonatal iron measures:										
Hemoglobin (g/dL) ^h^	-	-	0.22 †	0.22 †	-	-	−0.24 †	−0.09		
Ferritin (logged ug/L values) ^h^	-	-	0.26 †	0.22	-	-	-	-		
Hemoglobin (g/dL):log(ferritin) ^h^	-	-	0.26 †	0.21	-	-	-	-		
6.5-month infant iron measures:										
Hemoglobin (g/dL) ^h^	-	-	-	-	0.21 †	0.24 †	-	-		
Iron deficiency (Yes = 1, No = 0) ^g,i^	-	-	-	-	-	-	-	-		
Iron deficiency anemia (Yes = 1, No = 0) ^f,g,i^	-	-	-	-	-	-	0.23 †	0.24 †		
**Inflammation PCA Factor:**	**Factor 5**		**Factor 10 ^e^**		**Factor 12**		**Factor 22 ^d^**		**Factor 34 ^d^**	
	***r***	***ß***	***r***	***ß***	***r***	***ß***	***r***	***ß***	***r***	***ß***
Maternal iron measures:										
Ferritin (logged ug/L values) ^b^	-	-	−0.26 *	−0.23 *	-	-	-	-	-	-
Hemoglobin (g/dL):log(ferritin ug/L) ^b^	−0.22†	−0.19	0.39 ***	0.34 **	-	-	-	-	-	-
Urinary hepcidin (ng):creatinine (mg) ^g^	-	-	-	-	-	-	−0.31 †	−0.31	-	-
Neonatal iron measures:										
Hemoglobin (g/dL) ^h^	-	-	-	-	-	-	-	-	-	-
Ferritin (logged ug/L values) ^h^	-	-	-	-	-	-	-	-	-	-
Hemoglobin (g/dL):log(ferritin) ^h^	-	-	-	-	-	-	-	-	-	-
6.5-month infant iron measures:										
Hemoglobin (g/dL) ^h^	-	-	-	-	-	-	-	-	-	-
Iron deficiency (Yes = 1, No = 0) ^g,i^	-	-	-	-	0.22 †	0.23 †	0.30 **	0.27 *	-	-
Iron deficiency anemia (Yes = 1, No = 0) ^f,g,i^	-	-	-	-	0.27 *	0.26 *	-	-	-	-
**Inflammation PCA Factor:**	**Factor 35**		**Factor 40**		**Factor 45 ^6^**		**Factor 46**		**Factor 57 ^d,e^**	
	***r***	***ß***	***r***	***ß***	***r***	***ß***	***r***	***ß***	***r***	***ß***
Maternal iron measures:										
Ferritin (logged ug/L values) ^b^	-	-	-	-	-	-	-	-	-	-
Hemoglobin (g/dL):log(ferritin ug/L) ^b^	-	-	-	-	-	-	-	-	-	-
Urinary hepcidin (ng):creatinine (mg) ^g^	-	-	0.30 †	0.30 †	-	-	-	-	-	-
Neonatal iron measures:										
Hemoglobin (g/dL) ^h^	-	-	-	-	-	-	-	-	0.23 †	0.08
Ferritin (logged ug/L values) ^h^	-	-	-	-	-	-	-	-	0.24 †	0.12
Hemoglobin (g/dL):log(ferritin) ^h^	-	-	-	-	-	-	-	-	-	-
6.5-month infant iron measures:										
Hemoglobin (g/dL) ^h^	-	-	-	-	−0.23 †	−0.25 *	−0.20 †	−0.20	-	-
Iron deficiency (Yes = 1, No = 0) ^g,i^	-	-	-	-	-	-	-	-	-	-
Iron deficiency anemia (Yes = 1, No = 0) ^f,g,i^	-	-	-	-	-	-	-	-	-	-

† *p* ≤ 0.10; * *p* ≤ 0.05; ** *p* ≤ 0.01; *** *p* ≤ 0.001. *r* = Pearson correlation coefficient (N.B.: only values for which *p* < 0.10 are shown); *ß* = standardized regression coefficient adjusting for the time from delivery to placenta sample freezing, weeks’ gestation at delivery, and the following where noted: ^a^ no. prenatal cigarettes/day, ^b^ maternal age, ^c^ days/month prenatal marijuana use, ^d^ maternal education (highest grade completed), ^e^ socioeconomic status [23], ^f^ gravidity, ^g^ maternal iron supplementation (yes vs. no), ^h^ age at time of blood draw, and ^i^ number of weeks infant was formula fed. PCA = principal component analysis.

## Data Availability

De-identified, individual participant data that underlie the results reported in this article and the study protocol, statistical analysis plan, and analytic code will be available for sharing to journal editors for any reason either before or after publication for checking and to researchers who provide a methodologically sound proposal, as determined by the authors of this article. Proposals from interested parties should be directed to Sandra W. Jacobson, Ph.D. (sandra.jacobson@wayne.edu). The data will be stored in a data repository at Wayne State University and transmitted electronically in encrypted form to requestors. Data requestors will need to sign a data access agreement prior to access.

## Supplementary Materials

The following supporting information can be downloaded at https://www.mdpi.com/article/10.3390/nu15194105/s1: Supplementary File S1: Figure S1. Sample selection; Figure S2. Analysis plan; Figure S3. Principal component analysis scree plots; Table S1. Causal mediation analyses examining PCA factors as potential mediators of relations between prenatal alcohol exposure and maternal and infant iron outcomes; Supplementary S1. Gene function review of genes contributing to inflammation PCA factor 10; Supplementary File S2: Iron metabolism- and inflammation-related genes with MSigDB ontology terms and final gene sets; Supplementary File S3: Pearson correlations between prenatal alcohol exposure and PCA factors; PCA factors and maternal and infant iron outcomes; and individual genes and PCA factors. References [54,55,56,57,58] are cited in the supplementary materials.

## Abbreviations

AA = absolute alcohol; CRP = C-reactive protein; ERK = extracellular signal-regulated kinases; FAS = fetal alcohol syndrome; FASD = fetal alcohol spectrum disorders; FPN-1 = ferroportin-1; HIV = human immunodeficiency virus; ID = iron deficiency; IDA = iron deficiency anemia; IFN = interferon; IL = interleukin; JAK = Janus kinase; MAPK = mitogen-activated protein kinase; MSigDB = Molecular Signatures Database; NADPH = nicotinamide adenine dinucleotide phosphate; NF = nuclear factor; PCA = principal component analysis; PC = principal component analysis factor; RNA-Seq = ribonucleic acid sequencing; SES = socioeconomic status; SGA = small-for-gestational-age; STAT = signal transducers and activators of transcription; sTfR = soluble transferrin receptor; TFR-1 = transferrin receptor-1; TNF = tumor necrosis factor; UCT = University of Cape Town.

## References

[1] May P.A., Baete A., Russo J., Elliott A.J., Blankenship J., Kalberg W.O., Buckley D., Brooks M., Hasken J., Abdul-Rahman O. (2014). Prevalence and characteristics of fetal alcohol spectrum disorders. Pediatrics.

[2] Hoyme H.E., Kalberg W.O., Elliott A.J., Blankenship J., Buckley D., Marais A.-S., Manning M.A., Robinson L.K., Adam M.P., Abdul-Rahman O. (2016). Updated clinical guidelines for diagnosing fetal alcohol spectrum disorders. Pediatrics.

[3] Hoyme H.E., May P.A., Kalberg W.O., Kodituwakku P., Gossage J.P., Trujillo P.M., Buckley D.G., Miller J.H., Aragon A.S., Khaole N. (2005). A practical clinical approach to diagnosis of fetal alcohol spectrum disorders: Clarification of the 1996 Institute of Medicine criteria. Pediatrics.

[4] Popova S., Lange S., Probst C., Gmel G., Rehm J. (2017). Estimation of national, regional, and global prevalence of alcohol use during pregnancy and fetal alcohol syndrome: A systematic review and meta-analysis. Lancet Glob. Health.

[5] May P.A., Chambers C.D., Kalberg W.O., Zellner J., Feldman H., Buckley D., Kopald D., Hasken J.M., Xu R., Honerkamp-Smith G. (2018). Prevalence of Fetal Alcohol Spectrum Disorders in 4 US Communities. JAMA.

[6] May P.A., Blankenship J., Marais A.S., Gossage J.P., Kalberg W.O., Barnard R., De Vries M., Robinson L.K., Adnams C.M., Buckley D. (2013). Approaching the prevalence of the full spectrum of fetal alcohol spectrum disorders in a South African population-based study. Alcohol. Clin. Exp. Res..

[7] Carter R.C., Jacobson J.L., Molteno C.D., Jiang H., Meintjes E.M., Jacobson S.W., Duggan C. (2012). Effects of heavy prenatal alcohol exposure and iron deficiency anemia on child growth and body composition through age 9 years. Alcohol. Clin. Exp. Res..

[8] Carter R.C., Wainwright H., Molteno C.D., Georgieff M.K., Dodge N.C., Warton F., Meintjes E.M., Jacobson J.L., Jacobson S.W. (2016). Alcohol, methamphetamine, and marijuana exposure have distinct effects on the human placenta. Alcohol. Clin. Exp. Res..

[9] Oei J.L. (2020). Alcohol use in pregnancy and its impact on the mother and child. Addiction.

[10] Denny C.H., Acero C.S., Naimi T.S., Kim S.Y. (2019). Consumption of alcohol beverages and binge drinking among pregnant women aged 18–44 years-united states, 2015–2017. Morb. Mortal. Wkly. Rep..

[11] Carter R.C., Georgieff M.K., Ennis K.M., Dodge N.C., Wainwright H., Meintjes E.M., Duggan C.P., Molteno C.D., Jacobson J.L., Jacobson S.W. (2021). Prenatal alcohol-related alterations in maternal, placental, neonatal, and infant iron homeostasis. Am. J. Clin. Nutr..

[12] Ganz T. (2011). Hepcidin and iron regulation, 10 years later. Blood.

[13] Sangkhae V., Nemeth E. (2017). Regulation of the iron homeostatic hormone hepcidin. Adv. Nutr..

[14] Sangkhae V., Nemeth E. (2019). Placental iron transport: The mechanism and regulatory circuits. Free Radic. Biol. Med..

[15] Carter R.C., Jacobson S.W., Molteno C.D., Jacobson J.L. (2007). Fetal alcohol exposure, iron-deficiency anemia, and infant growth. Pediatrics.

[16] Bodnar T.S., Raineki C., Wertelecki W., Yevtushok L., Plotka L., Zymak-Zakutnya N., Honerkamp-Smith G., Wells A., Rolland M., Woodward T.S. (2018). Altered maternal immune networks are associated with adverse child neurodevelopment: Impact of alcohol consumption during pregnancy. Brain Behav. Immun..

[17] Sowell K.D., Uriu-Adams J.Y., Van de Water J., Chambers C.D., Coles C.D., Kable J.A., Yevtushok L., Zymak-Zakutnya N., Wertelecki W., Keen C.L. (2018). Implications of altered maternal cytokine concentrations on infant outcomes in children with prenatal alcohol exposure. Alcohol.

[18] Saini N., Helfrich K.K., Kwan S.T.C., Huebner S.M., Abazi J., Flentke G.R., Blohowiak S.E., Kling P.J., Smith S.M. (2019). Alcohol’s Dysregulation of Maternal-Fetal IL-6 and p-STAT3 Is a Function of Maternal Iron Status. Alcohol. Clin. Exp. Res..

[19] Ahluwalia B., Wesley B., Adeyiga O., Smith D.M., Da-Silva A., Rajguru S. (2000). Alcohol modulates cytokine secretion and synthesis in human fetus: An in vivo and in vitro study. Alcohol.

[20] Ohtake T., Saito H., Hosoki Y., Inoue M., Miyoshi S., Suzuki Y., Fujimoto Y., Kohgo Y. (2007). Hepcidin Is Down-Regulated in Alcohol Loading. Alcohol. Clin. Exp. Res..

[21] Carter R.C., Dodge N.C., Molteno C.D., Meintjes E.M., Jacobson J.L., Jacobson S.W. (2022). Mediating and moderating effects of iron homeostasis alterations on fetal alcohol-related growth and neurobehavioral deficits. Nutrients.

[22] Jacobson S.W., Chiodo L.M., Sokol R.J., Jacobson J.L. (2002). Validity of maternal report of prenatal alcohol, cocaine, and smoking in relation to neurobehavioral outcome. Pediatrics.

[23] Hollingshead A.B. (2011). Four factor index of social status. Yale J. Sociol..

[24] Beard J.L. (1994). Iron deficiency: Assessment during pregnancy and its importance in pregnant adolescents. Am. J. Clin. Nutr..

[25] Carriaga M.T., Skikne B.S., Finley B., Cutler B., Cook J.D. (1991). Serum transferrin receptor for the detection of iron deficiency in pregnancy. Am. J. Clin. Nutr..

[26] WHO, UNICEF, UNU (1998). Iron Deficiency Anemia: Prevention, Assessment and control—Report of a Joint WHO/UNICEF/UNU Consultation.

[27] Ganz T., Olbina G., Girelli D., Nemeth E., Westerman M. (2008). Immunoassay for human serum hepcidin. Blood.

[28] Stodgell C.J., Miller R.K., Salamone L., Murray J., Chen J., Lambertini L., Schadt E., Littman L., Landrigan P., Aagaard K. (2014). Lack of Correlation between placental gene expression and RNA integrity number (RIN) or time to collection. Placenta.

[29] Ewels P., Magnusson M., Lundin S., Käller M. (2016). MultiQC: Summarize analysis results for multiple tools and samples in a single report. Bioinformatics.

[30] Dobin A., Davis C.A., Schlesinger F., Drenkow J., Zaleski C., Jha S., Batut P., Chaisson M., Gingeras T.R. (2013). STAR: Ultrafast universal RNA-seq aligner. Bioinformatics.

[31] Hoffman G.E., Schadt E.E. (2016). variancePartition: Interpreting drivers of variation in complex gene expression studies. BMC Bioinform..

[32] Leek J.T., Johnson W.E., Parker H.S., Jaffe A.E., Storey J.D. (2012). The sva package for removing batch effects and other unwanted variation in high-throughput experiments. Bioinformatics.

[33] Subramanian A., Tamayo P., Mootha V.K., Mukherjee S., Ebert B.L., Gillette M.A., Paulovich A., Pomeroy S.L., Golub T.R., Lander E.S. (2005). Gene set enrichment analysis: A knowledge-based approach for interpreting genome-wide expression profiles. Proc. Natl. Acad. Sci. USA.

[34] Liberzon A., Birger C., Thorvaldsdóttir H., Ghandi M., Mesirov J.P., Tamayo P. (2015). The Molecular Signatures Database (MSigDB) hallmark gene set collection. Cell Syst..

[35] Ringnér M. (2008). What is principal component analysis?. Nat. Biotechnol..

[36] Stelzer G., Rosen N., Plaschkes I., Zimmerman S., Twik M., Fishilevich S., Stein T.I., Nudel R., Lieder I., Mazor Y. (2016). The GeneCards suite: From gene data mining to disease genome sequence analyses. Curr. Protoc. Bioinform..

[37] Pearl J., Breese J., Koller D. (2001). Direct and Indirect Effects.

[38] Robins J.M., Greenland S. (1992). Identifiability and exchangeability for direct and indirect effects. Epidemiology.

[39] First M.B., Williams J.B.W. (2021). Structured Clinical Interview for DSM-5 Disorders.

[40] Oken E., Kleinman K.P., Rich-Edwards J. (2003). A nearly continuous measure of birth weight for gestational age using a United States national reference. BMC Pediatr..

[41] Verga Falzacappa M.V., Vujic Spasic M., Kessler R., Stolte J., Hentze M.W., Muckenthaler M.U. (2007). STAT3 mediates hepatic hepcidin expression and its inflammatory stimulation. Blood.

[42] Wrighting D.M., Andrews N.C. (2006). Interleukin-6 induces hepcidin expression through STAT3. Blood.

[43] Miller A.M., Horiguchi N., Jeong W.I., Radaeva S., Gao B. (2011). Molecular mechanisms of alcoholic liver disease: Innate immunity and cytokines. Alcohol. Clin. Exp. Res..

[44] Bodnar T.S., Raineki C., Wertelecki W., Yevtushok L., Plotka L., Granovska I., Zymak-Zakutnya N., Pashtepa A., Wells A., Honerkamp-Smith G. (2020). Immune network dysregulation associated with child neurodevelopmental delay: Modulatory role of prenatal alcohol exposure. J. Neuroinflamm..

[45] Ruffaner-Hanson C., Noor S., Sun M.S., Solomon E., Marquez L.E., Rodriguez D.E., Allan A.M., Caldwell K.K., Bakhireva L.N., Milligan E.D. (2022). The maternal-placental-fetal interface: Adaptations of the HPA axis and immune mediators following maternal stress and prenatal alcohol exposure. Exp. Neurol..

[46] Young S.L., Saif Z., Meakin A.S., McMaster E.S., Hayes N., Gallo L.A., Reid N., Moritz K.M., Clifton V.L. (2021). Alterations to Placental Glucocorticoid Receptor Expression with Alcohol Consumption. Reprod. Sci..

[47] Teo S.M., Murrin C.M., Mehegan J., Douglass A., Hebert J.R., Segurado R., Kelleher C.C., Phillips C.M. (2023). Associations between the maternal healthy lifestyle score and its individual components during early pregnancy with placental outcomes. Placenta.

[48] Wang N., Tikellis G., Sun C., Pezic A., Wang L., Wells J.C.K., Cochrane J., Ponsonby A.L., Dwyer T. (2014). The effect of maternal prenatal smoking and alcohol consumption on the placenta-to-birth weight ratio. Placenta.

[49] Rufer E.S., Tran T.D., Attridge M.M., Andrzejewski M.E., Flentke G.R., Smith S.M. (2012). Adequacy of maternal iron status protects against behavioral, neuroanatomical, and growth deficits in fetal alcohol spectrum disorders. PLoS ONE.

[50] Rufer E.S., Tran T.D., Attridge M.E., Andrzejewski M.E., Smith S.M. (2010). Subclinical maternal iron inadequacy exacerbates neurobehavioral deficits caused by developmental ethanol exposure. Alcohol. Clin. Exp. Res..

[51] Mullarky I.K., Szaba F.M., Kummer L.W., Wilhelm L.B., Parent M.A., Johnson L.L., Smiley S.T. (2007). Gamma interferon suppresses erythropoiesis via interleukin-15. Infect. Immun..

[52] Lee P., Peng H., Gelbart T., Wang L., Beutler E. (2005). Regulation of hepcidin transcription by interleukin-1 and interleukin-6. Proc. Natl. Acad. Sci. USA.

[53] Kawasumi H., Gono T., Kawaguchi Y., Kaneko H., Katsumata Y., Hanaoka M., Kataoka S., Yamanaka H. (2014). IL-6, IL-8, and IL-10 are associated with hyperferritinemia in rapidly progressive interstitial lung disease with polymyositis/dermatomyositis. Biomed Res. Int..

[54] Safran M., Rosen N., Twik M., BarShir R., Iny Stein T., Dahary D. (2022). The GeneCards Suite. Practical Guide to Life Science Databases.

[55] Hayward P., Kalmar T., Arias A.M. (2008). Wnt/Notch signalling and information processing during development. Development.

[56] Guo Y.J., Pan W.W., Liu S.B., Shen Z.F., Xu Y., Hu L.L. (2020). ERK/MAPK signalling pathway and tumorigenesis. Exp. Ther. Med..

[57] Harrison D.A. (2012). The Jak/STAT pathway. Cold Spring Harb. Perspect. Biol..

[58] Seif F., Khoshmirsafa M., Aazami H., Mohsenzadegan M., Sedighi G., Bahar M. (2017). The role of JAK-STAT signaling pathway and its regulators in the fate of T helper cells. Cell Commun. Signal..

