# Supplementary material for: Alterations in Placental Inflammation-Related Gene Expression Partially Mediate the Effects of Prenatal Alcohol Consumption on Maternal Iron Homeostasis
Source: Nutrients. 2023 Sep 22;15(19):4105. doi: 10.3390/nu15194105 (PMC10574168; doi:10.3390/nu15194105)
Supplement: Supplementary file 1 [file nutrients-15-04105-s001.zip › nutrients-2586736-supplementary/Supplementary File S1_7 Sept 2023.docx]

**Supplementary File S1 for:**

Alterations in placental inflammation-related gene expression mediate effects of prenatal alcohol consumption on maternal iron homeostasis

Jacqueline Masehi-Lano, MS, Maya Deyssenroth, DrPH, Sandra W. Jacobson, PhD, Joseph L. Jacobson, PhD, Neil C. Dodge, PhD, Helen Wainwright, MB, ChB, Ernesta M. Meintjes, PhD, Corina Lesseur, MD, PhD, Haoxiang Cheng PhD, Qian Li, PhD, Ke Hao, PhD, Jia Chen, ScD, R. Colin Carter, MD, MMS


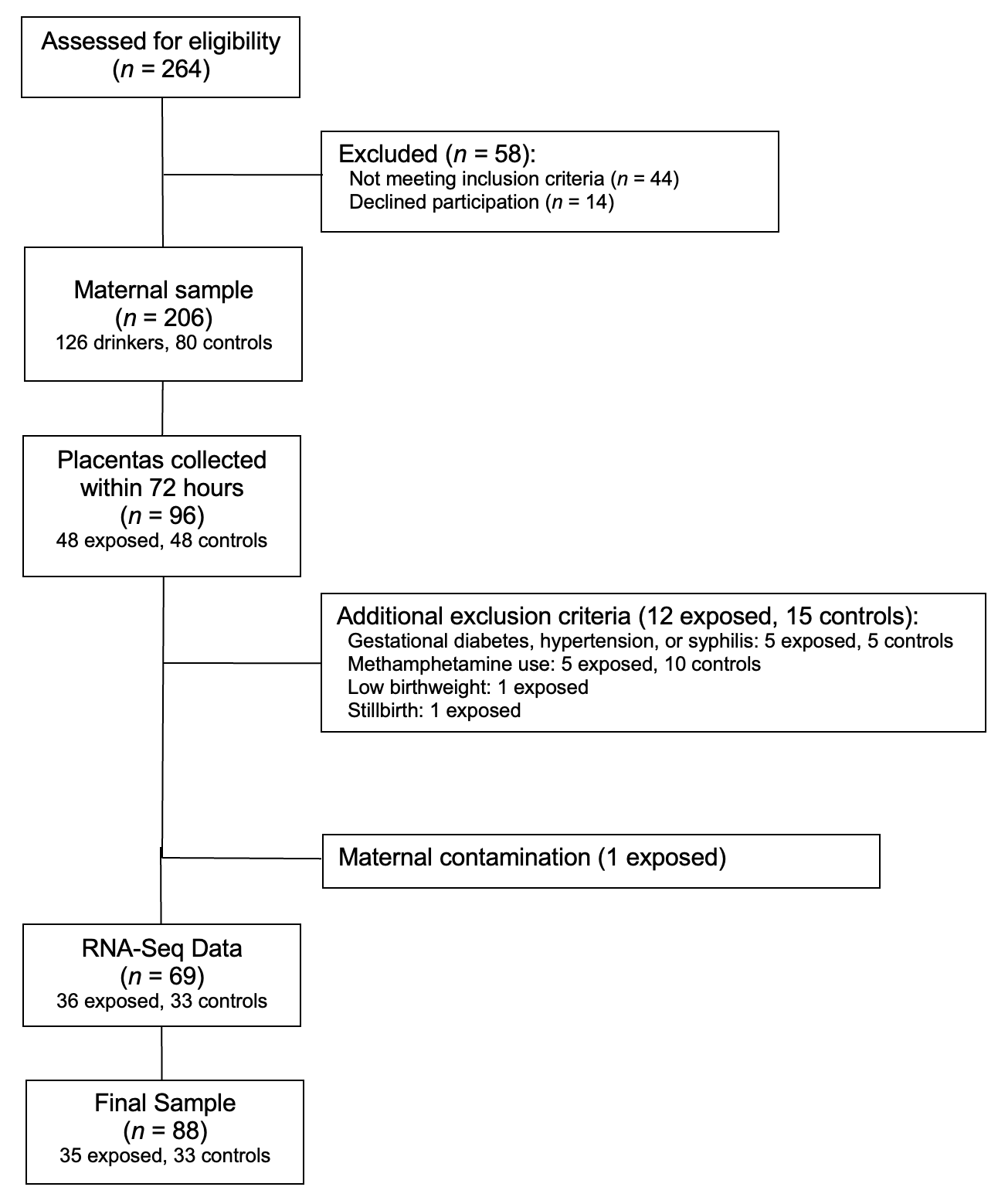


**Figure S1.** Sample selection.

Whole transcriptome RNA sequencing (RNA-seq) on bulk placental tissue

Gene set curation using iron metabolism and inflammation-related ontology terms in Molecular Signatures Database [1,2]

Principal component analysis (PCA) conducted on RNA expression data for each gene set

Association analyses between PCA factors and prenatal alcohol exposure (PAE) and maternal and infant iron outcomes

Causal inference analysis for variable triads where a PCA factor is related to PAE and a given iron outcome

Literature review on functions of genes contributing (|*r*| ≥0.2) to factors with evidence of mediation in causal inference analyses

**Figure S2.** Analysis plan.

1. Iron metabolism gene set


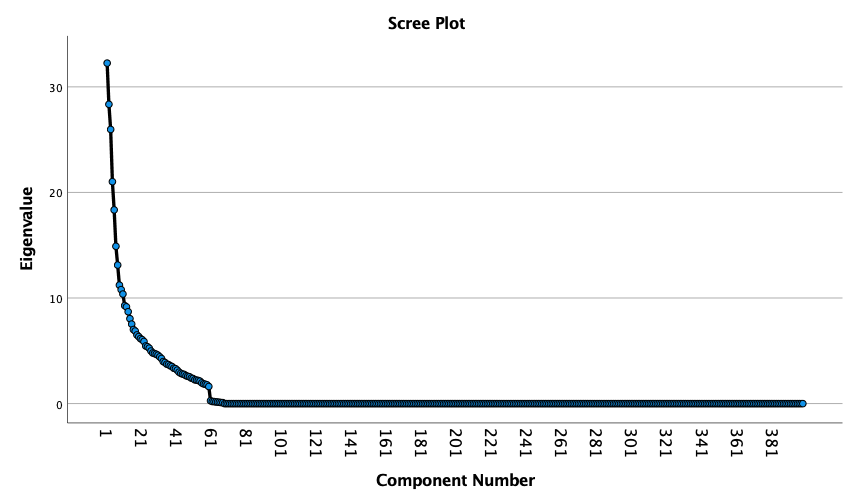


1. Inflammation-related gene set

**Figure S3.** Principal Component Analysis Scree Plots.

**Table S1. Causal mediation analyses examining PCA factors as potential mediators for relations between prenatal alcohol exposure and maternal and infant iron outcomes.**

|  | Model 1  (direct effect) | Model 2 | Model 3 | | Natural  indirect effect | Total effects |  |
| --- | --- | --- | --- | --- | --- | --- | --- |
|  | B_alc_ | B_alc_ | B_alc_ | B_fac_ | B | B | Proportion mediation^a^ |
| Iron PCA factor 18 |  |  |  |  |  |  |  |
| Relation of drinking days per week across pregnancy to 6.5-month hemoglobin with mediator PC18^a,b^ | 0.04  (-0.83, 0.91) | -0.27*  (-0.52, 0.01) | 0.01  (-0.28, 0.31) | 0.35*  (0.07, 0.64) | -0.28  (-0.64, 0.06) | -0.25  (-1.13, 0.64) | -- |
| Inflammation PCA Factor 10 |  |  |  |  |  |  |  |
| Relation of drinking days per week around conception to maternal ferritin (logged values) with mediator PC10^c,d^ | 0.16  (-0.22, 0.53) | -0.24*  (-0.42, -0.05) | 0.05  (-0.07, 0.18) | -0.11  (-0.27, 0.05) | 0.08  (-0.05, 0.21) | 0.23  (-0.12, 0.59) | -- |
| Relation of drinking days per week around conception to maternal hemoglobin:log(ferritin) with mediator PC10)^c,d^ | -0.18  (-0.64, 0.28) | -0.24*  (-0.42, -0.05) | -0.06  (-0.21, 0.10 | 0.27*  (0.06, 0.47) | -0.19^†^  (-0.39, 0.01) | -0.37  (-0.84, 0.09) | 51.35% |
| Inflammation PCA Factor 12 |  |  |  |  |  |  |  |
| Relation of drinking days per week around conception to infant iron deficiency anemia at age 6.5 months with mediator PC12^e,f,g^ | 0.12  (-0.16, 0.42) | 0.19^†^ (-0.02, 0.40) | 0.04  (-0.06, 0.13) | 0.09  (-0.04, 0.21) | 0.05  (-0.04, 0.14) | 0.17  (-0.11, 0.45) | -- |
| Inflammation PCA Factor 45 |  |  |  |  |  |  |  |
| Relation of average oz absolute alcohol/day (logged values) around conception to 6.5-month hemoglobin with mediator PC45^b,e^ | -0.06  (-0.53, 0.40) | 0.29  (-0.22, 0.80) | -0.08  (-0.68, 0.52) | 0.01  (-0.29, 0.30) | 0.00  (-0.06, 0.07) | -0.06  (-0.52, 0.40) | -- |

Values are B (95% CI) from marginal structural models using the product method [3,4].

Model 1 = relation of alcohol measure to outcome, independent of the mediator; Model 2 = PCA factor regressed on alcohol measure; Model 3 = outcome regressed on alcohol measure, the PCA factor.

B_alc_ = regression coefficient for alcohol measure; B_fac_ = regression coefficient for the PCA factor.

^a^Natural indirect effect/total effects

All models include covariates time from delivery to placenta sample freezing, weeks gestation at delivery, and the following where noted: ^a^maternal education (highest grade completed), ^b^age at time of blood draw, ^c^maternal age, ^d^socioeconomic status [5], ^e^gravidity, ^f^maternal iron supplementation (yes vs. no), ^g^number of weeks infant was formula fed, ^h^age at time of blood draw.

**Supplementary S4. Functions of** **genes related to inflammation PCA factor 10 at |*r*| >0.20**

(Except where otherwise cited, all information below is taken from GeneCards - the human gene database [6])

*Immune Cell Regulation*

Of the 48 placental genes involved in immune cell regulation that were positively associated with inflammation PCA factor 10, 5 functioned in immune cell activation (*IL1A, IL1B, FFAR2, S100A9, SLC7A2*), 4 were involved in immune cell differentiation (*EVI2B,* *IL1B, LOXL3, TESC*), 10 were linked to immune cell chemotaxis and adhesion (*CCL2, CCL28, CXCL8, FFAR2, S100A8, S100A9, PF4, PPBP, SELL, VAV3*), 4 played critical roles in phagocytosis (*IL15, S100A8, S100A9, VAV3*), and 3 participated in NADPH oxidase activation (*NCF1, S100A8, S100A9*), which is necessary for superoxide production. Within the 41 placental genes involved in immune cell regulation that were negatively associated with the factor: 11 functioned in immune cell activation (T-lymphocyte activation: *CD6, CD8A, CD44, IL7R, NFATC1, NFATC2, STAT3*; activation of other immune cell types: *CCL5, CXCL9, HLA-E, ZBTB46*), 8 were involved in immune cell chemotaxis and adhesion (*CCL5, CD44, CD6, CMKLR1, CXCL16, CXCL9, PECAM1, TEM*), 1 played a role in phagocytosis (*PECAM1*), and 1 participated in NAPDH oxidase activation during neutrophil differentiation (*LBR*). *AKT* is particularly relevant in its role as a regulator of placental development, and *HLA-E* binds to the receptors on natural killer (NK) cells with effects on maternal-fetal immune tolerance during pregnancy. Genes in the proinflammatory NF-κB signaling pathway, which activates the transcription of a large array of genes involved in immune reaction, growth regulation, and protection against apoptosis, also varied in their relations to the factor: *RAF1* and *RIPK2*, which are positively related to inflammation PCA factor 10, are potent activators of NF-κB and *CMKLR1*, which is negatively related to the factor, takes part in the negative regulation of NF-κB transcription factor activity.

*Cytokines*

*IL1A*, *IL1B*, *CXCL8*, and *IL15* were among the genes encoding cytokines IL-1α, IL-1β, IL-8, and IL-15, respectively, that are induced as an inflammatory response to increased drinking frequency around the time of conception. *ITGA5*, encoding a receptor for IL-1β that is essential for IL-1β signaling; *MEFV*, which acts as both an immune sensor that triggers IL-1β and IL-18 production while preventing their excess; *CXCR2*, encoding a receptor for IL-8; and *FOS*, a regulator of TGF-β-mediated signaling were also found to be positively associated with inflammation PCA factor 10. Genes related to interferons, which are cytokines produced in response to infections, were positively related to the factor: *IL1B* plays an additional role in synergizing with IL-12 to induce the synthesis of IFN-γ, and *MEFV* is involved in the inflammatory response to IFN-γ. Other genes related to cytokine activity, including *A2M, IL20RB*, *IL6ST*, *IL7R,* were negatively associated with inflammation PCA factor 10. *NFATC2*, which is a transcription factor that induces the expression of cytokine genes encoding IL-2, IL-3, IL-4, TNF-α, and granulocyte-macrophage colony-stimulating factor (GM-CSF) in T-cells, was also found to be negatively related to the factor.

*Angiogenesis and Hematopoiesis*

Angiogenesis and hematopoiesis represented two prominent categories of genes comprising inflammation PCA factor 10. Within the placental genes involved in hematopoiesis (*n* = 4) that were positively associated with the factor, *EVI2B* is required for the survival and functionality of hematopoietic progenitor cells by regulating cell cycle progression, *L3MBTL3* is a regulator of hematopoiesis, and both *TAL1* and *STAT5B* are involved in hemopoietic and erythrocyte differentiation. Three genes related to hematopoiesis and angiogenesis were negatively associated with the factor: *IL6ST*, encoding a protein involved in embryonic development and mediation of signals regulating hematopoiesis; *INHBA*, encoding a subunit of the dimeric inhibin and activin protein complexes that play a role in erythroid differentiation and germ cell development; and *APLNR*, which influences the position of future blood formation during early development.

*Ubiquitous Cell Functions*

Of the placental genes positively related to the factor, 6 were involved in cell division, proliferation, and differentiation (*FOS, GPC3, IL1B, PF4, PPBP, RAF1*); and 6 functioned in apoptosis-promoting or -inhibiting pathways (*GPC3, IL15, IL1B, RIPK2, STAT5B, RAF1*). A smaller number of placental genes involved in ubiquitous cell functions were negatively related to the factor, including *STAT3*, which is involved in cell cycle regulation; *EMP3*, which plays a role in cell proliferation; and *CXCL16*, which is linked to the positive regulation of cell growth.

Placental genes involved in the evolutionarily conserved Notch, Wnt, MAPK/ERK, and JAK/STAT signaling pathways also varied in their relations to the factor. Notch and Wnt are intracellular signaling pathways that are essential for early developmental processes such as cell fate decisions during embryonic development and hematopoiesis [7]. Two placental genes involved in these pathways were positively associated with inflammation PCA factor 10: *GPC3*, a positive regulator of both the canonical and non-canonical Wnt signaling pathways, and *PSEN1*, which plays roles in the Notch and Wnt signaling cascades as well as in regulating downstream pathways. The placental gene *IL6ST*, an activator of the Notch pathway for the regulation of epithelial regeneration induced by inflammation, showed a negative association with the factor. The MAPK/ERK signaling cascade is involved the transcriptional and translational regulation of numerous cell processes such as cell growth, division (including mitosis and meiosis), proliferation, differentiation, apoptosis, angiogenesis and the stress response [8]. ERK is a type of serine/threonine protein kinase and part of the MAPK family. Located in the cytoplasm, ERK transmits extracellular signals to intracellular targets by translocating to the nucleus upon activation and regulating transcription and gene expression. Four placental genes related to this signaling cascade were found to be positively related to inflammation PCA factor 10 (*CD40, RAF1, S100A9, TESC*). Three of these genes are involved in the MAPK/ERK cascade activation with regards to the immune response: *CD40* aids in the activation of ERK in macrophages and B cells for immunoglobulin secretion, *S100A9* induces MAPK-dependent neutrophil granulation, and *TESC* functions in ERK cascade activation and ERK-dependent granulocytic differentiation. *RAF1* is an essential link between Ras GTPases and the MAPK/ERK cascade for the regulation of cell fate specification, proliferation, differentiation, and apoptosis. Within the group of placental genes negatively related to inflammation PCA factor 10, the phosphorylation of *MAPK1*, also known as *ERK2*, can lead to diminished immune response and enhancement of angiogenesis and adipogenesis. The JAK/STAT pathway plays a role in the cellular response to cytokine and growth factor stimulation, resulting in the activation or suppression of the transcription of genes required for cell proliferation, differentiation, and apoptosis [9,10]. These cell functions are necessary for organismal developmental processes such as growth, hematopoiesis, immune system development, and mammary gland development. The JAK/STAT pathway is also essential for the differentiation of T helper cells [10]. Three genes associated with the JAK/STAT pathway exhibited a positive association with inflammation PCA factor 10: *IL15*, which activates transcription factors STAT3, STAT4, and STAT6; *LOXL3*, an inhibitor of STAT3 transcription activity; and *STAT5B*, a positive regulator of hematopoietic and erythroid differentiation. When phosphorylated by receptor-associated kinases, members of the STAT family are translocated to the nucleus and act as transcription activators. *STAT3*, encoding its namesake transcription activator STAT3 that induces hepatic hepcidin expression during inflammation, was unexpectedly negatively related to the factor [11,12].

**References**

1. Subramanian, A.; Tamayo, P.; Mootha, V.K.; Mukherjee, S.; Ebert, B.L.; Gillette, M.A.; Paulovich, A.; Pomeroy, S.L.; Golub, T.R.; Lander, E.S., et al. Gene set enrichment analysis: a knowledge-based approach for interpreting genome-wide expression profiles. *Proc Natl Acad Sci U S A* **2005**, *102*, 15545-15550, doi:10.1073/pnas.0506580102.

2. Liberzon, A.; Birger, C.; Thorvaldsdóttir, H.; Ghandi, M.; Mesirov, J.P.; Tamayo, P. The Molecular Signatures Database (MSigDB) hallmark gene set collection. *Cell Syst* **2015**, *1*, 417-425, doi:10.1016/j.cels.2015.12.004.

3. Pearl, J. *Direct and indirect effects*; Breese, J., Koller, D., Eds.; Morgan Kaufmann Publishers Inc.: San Francisco (CA), 2001.

4. Robins, J.M.; Greenland, S. Identifiability and exchangeability for direct and indirect effects. *Epidemiology* **1992**, *3*, 143-155, doi:10.1097/00001648-199203000-00013.

5. Hollingshead, A.B. Four factor index of social status. *Yale J Sociol* **2011**, *8*, 21-51.

6. Safran M, R.N., Twik M, BarShir R, Iny Stein T, Dahary D, Fishilevich S, and Lancet D. The GeneCards Suite Chapter, Practical Guide to Life Science Databases. 2022; pp 27-56.

7. Hayward, P.; Kalmar, T.; Arias, A.M. Wnt/Notch signalling and information processing during development. *Development* **2008**, *135*, 411-424, doi:10.1242/dev.000505.

8. Guo, Y.J.; Pan, W.W.; Liu, S.B.; Shen, Z.F.; Xu, Y.; Hu, L.L. ERK/MAPK signalling pathway and tumorigenesis. *Exp Ther Med* **2020**, *19*, 1997-2007, doi:10.3892/etm.2020.8454.

9. Harrison, D.A. The Jak/STAT pathway. *Cold Spring Harb Perspect Biol* **2012**, *4*, doi:10.1101/cshperspect.a011205.

10. Seif, F.; Khoshmirsafa, M.; Aazami, H.; Mohsenzadegan, M.; Sedighi, G.; Bahar, M. The role of JAK-STAT signaling pathway and its regulators in the fate of T helper cells. *Cell Commun Signal* **2017**, *15*, 23, doi:10.1186/s12964-017-0177-y.

11. Verga Falzacappa, M.V.; Vujic Spasic, M.; Kessler, R.; Stolte, J.; Hentze, M.W.; Muckenthaler, M.U. STAT3 mediates hepatic hepcidin expression and its inflammatory stimulation. *Blood* **2007**, *109*, 353-358, doi:10.1182/blood-2006-07-033969.

12. Wrighting, D.M.; Andrews, N.C. Interleukin-6 induces hepcidin expression through STAT3. *Blood* **2006**, *108*, 3204-3209, doi:10.1182/blood-2006-06-027631.
